# Statistical Experimental Design Guided Optimization of a One-Pot Biphasic Multienzyme Total Synthesis of Amorpha-4,11-diene

**DOI:** 10.1371/journal.pone.0079650

**Published:** 2013-11-20

**Authors:** Xixian Chen, Congqiang Zhang, Ruiyang Zou, Kang Zhou, Gregory Stephanopoulos, Heng Phon Too

**Affiliations:** 1 Chemical and Pharmaceutical Engineering, Singapore-MIT Alliance, Singapore, Singapore; 2 Department of Biochemistry, National University of Singapore, Singapore, Singapore; 3 Department of Chemical Engineering, Massachusetts Institute of Technology, Cambridge, Massachusetts, United States of America; Dowling College, United States of America

## Abstract

*In vitro* synthesis of chemicals and pharmaceuticals using enzymes is of considerable interest as these biocatalysts facilitate a wide variety of reactions under mild conditions with excellent regio-, chemo- and stereoselectivities. A significant challenge in a multi-enzymatic reaction is the need to optimize the various steps involved simultaneously so as to obtain high-yield of a product. In this study, statistical experimental design was used to guide the optimization of a total synthesis of amorpha-4,11-diene (AD) using multienzymes in the mevalonate pathway. A combinatorial approach guided by Taguchi orthogonal array design identified the local optimum enzymatic activity ratio for Erg12:Erg8:Erg19:Idi:IspA to be 100∶100∶1∶25∶5, with a constant concentration of amorpha-4,11-diene synthase (Ads, 100 mg/L). The model also identified an unexpected inhibitory effect of farnesyl pyrophosphate synthase (IspA), where the activity was negatively correlated with AD yield. This was due to the precipitation of farnesyl pyrophosphate (FPP), the product of IspA. Response surface methodology was then used to optimize IspA and Ads activities simultaneously so as to minimize the accumulation of FPP and the result showed that Ads to be a critical factor. By increasing the concentration of Ads, a complete conversion (∼100%) of mevalonic acid (MVA) to AD was achieved. Monovalent ions and pH were effective means of enhancing the specific Ads activity and specific AD yield significantly. The results from this study represent the first *in vitro* reconstitution of the mevalonate pathway for the production of an isoprenoid and the approaches developed herein may be used to produce other isopentenyl pyrophosphate (IPP)/dimethylallyl pyrophosphate (DMAPP) based products.

## Introduction

Malaria is a contagious disease that has claimed millions of life annually, and continues to infect more than 0.5% global population, especially in less developed nations [1]. Artemisinin is the key ingredient of the most potent treatment to Malaria [2]. It belongs to the diverse class of isoprenoids that are derived from the building blocks: IPP and DMAPP. Traditional supply of artemisinin solely depended on extraction from the leaves of the sweet wormwood plant *Artemisia annua* [3]. Since the growth of crops is slow and seasonal, this method inevitably results in supply fluctuation of artemisinin [4]. Efforts in metabolic engineering and synthetic biology have made some promises to even out the supply cycle by engineering fast growing microbes to produce artemisinin and its precursor, artemisinic acid [5]. Recently, a multistep semi-synthesis of artemisin has been reported where yeast cell was used to produce precursors for further chemical conversions [6]. Invariably, the complex cellular environment renders any optimization process a challenge to control and maximize productivity [7].

*In vitro* multienzyme pathway assembly is a useful approach complementing *in vivo* metabolic engineering [8]. Cheng et al. demonstrated the feasibility of producing polyketide by enzymatic total synthesis [9]. They assembled 12 pathway enzymes from different production hosts and were able to achieve an overall yield of 25% from simple raw material. This bottom-up method successfully bypasses cellular barriers and allows a higher degree of freedom for pathway manipulation. At the same time, it still ensures regioselectivity and enantioselectivity of the product. Moreover, enzymatic reactions involve fewer chemicals that can simplify purification and reduce the cost of downstream process. *In vitro* multienzyme biosynthesis has been touted as a promising technology that may replace many chemical synthesis processes due to its high efficiency [10].

**Figure 1:**
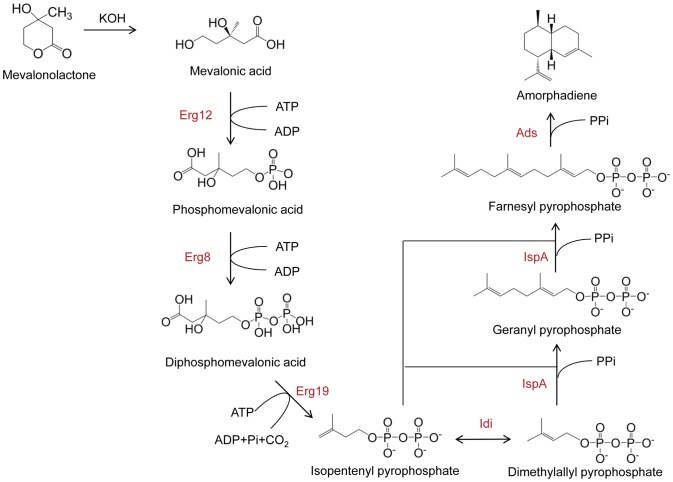
Mevalonate pathway for amorpha-4,11-diene production. The abbreviations are as follows. Erg12: mevalonate kinase, Erg8: phosphomevalonate kinase, Erg19: diphosphomevalonate decarboxylase, Idi: isopentenyl pyrophosphate isomerase, IspA: farnesyl pyrophosphate synthase, Ads: amorpha-4,11-diene synthase, Pi: phosphate, Ppi: pyrophosphate.

To our best knowledge, the production of artemisinin or its precursors *in vitro* is yet to be explored. Here we demonstrated the synthesis of amorpha-4,11-diene (AD), a key precursor to artemisinin, from mevalonic acid (MVA) by assembling seven enzymatic steps in one-pot with two-phase reaction condition (Figure 1). Previous *in vivo* analysis has shown that pathway balancing is critical to maximize the production of scarce therapeutic products [11], [12]. Therefore, we set to optimize the pathway productivity by means of Taguchi orthogonal array design in an attempt to balance the enzymatic levels under pre-determined reaction conditions. The information gained led us to identify an inhibitory step of farnesyl pyrophosphate synthase (IspA), the critical factor Ads and significantly improved the AD yield. Therefore, this technique developed in the present study demonstrated a complementary way of producing valuable drug precursors with the ability to identify the limiting steps and balance the enzymatic flux in an efficient manner.

## Materials and Methods

### Bacteria Strains and Plasmids

Bacteria strains and plasmids used in this study were summarized in Table S1. The pET-11a (Stratagene, CA) was modified by replacing the T7 promoter with tac promoter to facilitate the transfer of the plasmids among different strains. A 5′ SacI site and a 3′ XhoI site were introduced downstream from the 6xHis open reading frame. The mevalonate pathway enzymes, namely mevalonate kinase (Erg12), phosphomevalonate kinase (Erg8) and pyrophosphomevalonate decarboxylase (Erg19), were amplified from *S. cerevisiae* genomic DNA with forward and reverse primers that contain corresponding SacI and XhoI sites. The PCR products were ligated into the modified pET-11a vector (Stratagene, CA) and transformed into competent *E. Coli* strain DH10B. Isopentenyl pyrophosphate isomerase (Idi) and IspA were from our previous study [13]. Ads gene was codon optimized and synthesized by Genescript with sequences encoding C-terminal 6xHis-tag, and subsequently cloned into a modified pBAD-B vector (Invitrogen, CA) using 5′ SacI site and 3′ XhoI site. The primers used for amplification of the genes were listed in Table S2. All the plasmids were transformed and harboured from *E. coli* XL10-gold (Stratagene, CA) and then transformed to respectively strains for enzyme overexpression (Table S1).

### Expression and Purification of Erg12, Erg8, Erg19, Idi and IspA

Newly transformed colonies were picked from the agar plate, inoculated into 2xPY medium (20 g/L Peptone, 10 g/L Yeast extract, and 10 g/L NaCl, pH 7) containing 100 mg/L ampicillin and grew till stationary phase overnight at 37°C in an incubator-shaker (Shin Saeng Shaking Incubator, Finetech, Korea). The culture was then further transferred into fresh 2xPY medium (1% inoculation) with ampicillin for another 2.5 h at 37°C, till optical density A_600_ reached 0.6–1.0. The enzyme expression was induced with 0.1 mM isopropyl-1-thio-β-D-galactopyranoside (IPTG). Temperature was reduced to 20°C after induction for higher solubility of the enzymes [13]. The culture was grown for another 48 h and harvested by centrifugation. The cell pellets were stored at −20°C till further use.

To purify the enzymes, the frozen cell pellets were resuspended in B-PERII reagent (Pierce, IL), according to the manufacturer’s instruction, and vortexed at room temperature for 30 mins to completely lyse the cells. The soluble proteins were contained in the supernatant, which was diluted 15 times in NPI10 buffer (50 mM NaH_2_PO_4_, 300 mM NaCl, 10 mM imidazole, pH 8) and incubated with 200 mg Ni-NTA resin (USB, Affymetrix, CA) at 4°C for 2 h. The resin was washed with NPI10 buffer after discarding the binding supernatant, and the enzymes were eluted and collected by 400 µl NPI400 (50 mM NaH_2_PO_4_, 300 mM NaCl, 400 mM imidazole, pH 8). The enzymes were further concentrated by 3K Amicon ultra-0.5 ml centrifugal filter unit (Millipore, MA), and the concentrations were measured by Micro BCA protein assay kit (Thermo scientific, MA). The purified enzymes were further confirmed by sodium dodecyl sulfate-12% polyacrylamide gel electrophoresis (Bio-Rad, CA).

### Expression and Purification of Ads

Bacteria culture was grown in 2xPY medium at 20°C till stationary phase after Ads expression was induced with 10 mM L-arabinose. The cells were harvested by high speed centrifugation and resuspended in phosphate saline buffer (PBS). To purify Ads, cells were lysed by three rapid freeze-thaw cycles by −80°C freezer and 37°C incubator. The released enzyme was separated from cell debris by centrifuging at 3000 *g* for 15 min, and purified by Ni-NTA resin as described above.

### Enzyme Kinetics

The pathway enzyme kinetics was determined individually by initial rate measurements. In brief, the substrates and cofactors were added to 100 mM Tris/HCl reaction buffer (pH 7.4), and the reaction was initiated by adding pre-determined enzyme amount to ensure less than 10% substrate was consumed in 15 mins at 30°C. The substrates concentrations were varied in equal steps in reciprocal space from 0.1 to 1 mM. The reaction was terminated by adding equal volume of 1% ammonium hydroxide and diluted 10 times into cold methanol. After high speed centrifugation, the supernatant was subject to UPLC-(TOF)MS analysis. Double-reciprocal plots of each enzymatic activity were constructed for the determination of K_m_ and K_cat_ values for the respective substrates.

### Multienzyme Reaction

The multienzyme reaction was carried out in a buffer (25 µl) consisted of Tris/HCl (100 mM, pH 7.4), MgCl_2_ (10 mM), (±)mevalonic acid (10 mM), ATP (15 mM) and the purified enzymes. The reaction was performed at 30°C with an overlay of dodecane phase that contained trans-caryophyllene (50 mg/L) as an internal standard. At the end of the reaction, the dodecane phase was diluted 10 times in ethyl acetate and subject to GCMS analysis. The (±)mevalonic acid was prepared by complete alkaline hydrolysis of 2 M (±)mevalonolactone (Sigma, MO) with equal volume of 2 M potassium hydroxide at 37°C for 1.5 h, and neutralized by adding 1 M hydrochloric acid to pH 7 [14].

### Experimental Design

Taguchi orthogonal array design and response surface methodology with central composite design were calculated using Design Expert® V8 Software (Stat-Ease, Inc). Taguchi L_16_ (4^5^) orthogonal array was constructed which can accommodate five control factors corresponding to the five pathway enzymes, each varied at four levels of concentrations (Table S3). The four enzymatic levels were normalized against Ads activity (AA), ranging from 1xAA, 5xAA, 25xAA and 100xAA to achieve sufficient coverage. The lowest level was equalized enzymatic activity, whereas the highest level was comparable enzymatic concentrations. The level of Idi was varied according to IspA. 16 randomized experimental runs were conducted to maximize AD yield (Equation 1). The specific AD yield (Equation 2) was another indicator of the pathway productivity but was not considered in the design experiment. The two dimensionless readouts were calculated as follows:
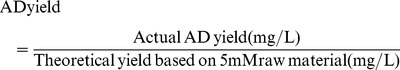
(1)

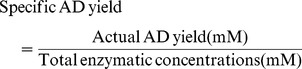
(2)


Optimizing IspA and Ads activities was carried out by response surface methodology with central composite design, which involved the investigation of two factors (concentrations of IspA and Ads), each varied at five levels and four centre points for replication. The AD production at 6 h was taken as the response, before the reaction reached completion and any visible precipitations were formed. The experimental data obtained were fitted based on the most suitable model suggested by the software.

### UPLC-(TOF)MS Analysis of Mevalonate Pathway Intermediates

The analysis was done based on the method developed previously with slight modification [15]. In brief, 5 µl samples were injected into a UPLC C18 column (Waters CSH C18 1.7 µm, 2.1 mm x 50 mm) connected to UPLC (Waters ACQUITY UPLC)-(TOF)MS (Bruker micrOTOF II, MA). Elution was carried out with a step change from 100% aqueous solution containing 15 mM acetic acid and 10 mM tributylamine (0.5 min) to 10% aqueous solution with 90% methanol for another 3.5 min. Electrospray ionization was used and mass spectrometry was operated to scan 50–800 m/z in negative mode with 2500 V end plate voltage and 3200 V capillary voltage. Nebulizer gas was provided in 2 bar, dry gas flow rate was 9 ml/min, and dry gas temperature was 200°C. At the assay condition, all the intermediates were detected in the form [M-H]^−^. Retention time was subsequently determined for each intermediate with respective synthetic standards and the set m/z extraction range. The peak area was calculated and subsequently used to compute the intermediates concentrations with the software provided by the manufacturer. The calibration curves were constructed with synthetic standards prepared under similar reaction conditions without enzymes. Linearity of the assays were determined individually with coefficients of determinants (R^2^) greater than 0.90.

### GCMS Analysis of Amorpha-4,11-diene

The analysis was carried out based on the modified method developed by Martin et al. by scanning three ions; the m/z values are 117, 189 and 204 [14], [16]. 1 µl sample was injected into HP-5 column (Agilent Technologies 7890A gas chromatograph-mass spectrometry, Agilent, CA) with a linear temperature increase of 50°C/min from 80°C to 300°C and hold at 300°C for another minute. The peak area was calculated and subsequently used to compute the amorpha-4,11-diene concentrations with the software provided by the manufacturer. Amorpha-4,11-diene concentrations were determined relative to the internal standard trans-caryophyllene of known concentration.

## Results

### Enzymatic Purification and Characterization

Individual enzyme was overexpressed in *E. coli* strains. Sodium dodecylsufate polyacrylamide gel electrophoresis (SDS-PAGE) results showed that the enzymes were expressed at high levels. However, the yield of purified individual enzymes obtained by immobilized metal affinity chromatography differed significantly (Table 1). This was mainly due to the differences in the solubility of the enzymes (Figure S1) [13]. In particular, there was almost no detectable soluble fraction of Ads (Figure S1). This led us to extensively optimize the strains, growth conditions and enzyme extraction methods for Ads. Repeated freeze-thaw method [17] was found to be effective in isolating Ads (1.6 mg/L) from cells with high purity as compared to detergent based lysis method. An initial attempt was made by mixing equal mass of the six enzymes in one pot with an overlay of dodecane phase where amorpha-4,11-diene was found to be produced in trace amounts (results not shown).

**Table 1:** Purification and characterizations of individual pathway enzymes from bacterial culture.

Enzyme	Synonyms	EC #	MW**	K_m_/µM	K_cat_/s^−1^	Enzyme Yield*/mg/L
Mevalonate kinase	Erg12	2.7.1.36	49524	460±153 (MVA)	5.5±1.6	2–8
Phosphomevalonate Kinase	Erg8	2.7.4.2	51520	780±280 (PMVA)	22.0±7.0	1.5–2.5
Diphosphomevalonate Decarboxylase	Erg19	4.1.1.33	45181	190±52 (PPMVA)	2.8±0.5	15–60
Isopentyl pyrophosphate isomerase	Idi	5.3.3.2	21331			6.5–28
Farnesyl pyrophosphate synthase	IspA	2.5.1.92	32982	200±92 (IPP)	1.5±0.6	0.75–2
Amorpha-4,11-diene synthase	Ads	4.2.3.24	64624	43.72±10.4	0.05±0.013	0.3–1.6

The bracket contains the specific substrate that the K_m_ is measured for.

* The enzyme yield is defined as the final amount of enzymes obtained after purification from a liter bacterial culture. The results have been repeated for more than three times.

** The molecular weight (MW) of the enzyme was calculated based on its amino acid sequence.

In order to better understand and optimize the *in vitro* system, steady-state kinetics of each enzyme was initially measured. The results were summarized in Table 1. The enzymatic concentrations were determined to ensure the measurement of the initial rate of reaction were linear in the first 15 mins. From the results, Ads displayed a significantly lower turnover number, a 2 orders of magnitude lower than the other five enzymes. It seemed to be an intrinsic property of terpene synthases, which was proposed to be limited by the release of the product [18]–[20]. Therefore, Ads was identified to be the bottleneck step in the multienzyme synthesis reaction.

### Tuning Enzymatic Levels by Taguchi Orthogonal Array Design

**Figure 2:**
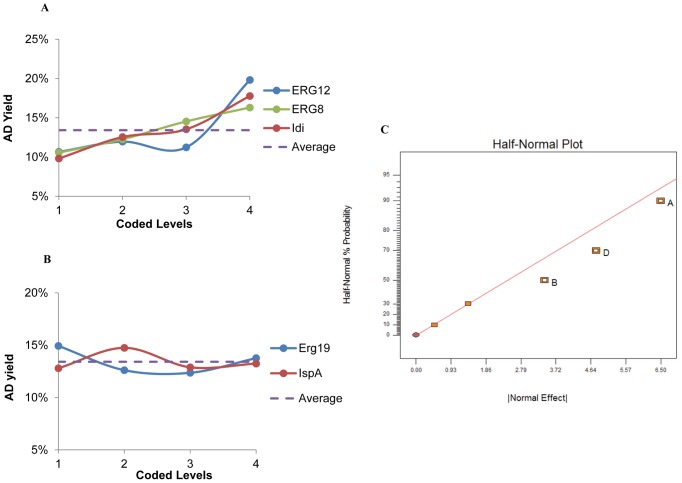
The Taguchi orthogonal array design results. To examine the influence of each enzyme on amopha-4,11-diene (AD) yield, the average effects analysis was determined. The five enzymes can be classified into two main groups. A: Average values of each level of factors Erg12, Erg8 and Idi on AD yield. The group of enzymes has a positive correlation with AD yield. B: Average values of each level of factors Erg19 and IspA on AD yield. The group of enzymes has little or no effect on AD yield. C: The half-normal plot indicates the significant factors on AD yield. Factor A, B, D represent Erg12, Erg8 and Idi respectively. The abbreviations are as follows. Erg12: mevalonate kinase, Erg8: phosphomevalonate kinase, Erg19: diphosphomevalonate decarboxylase, Idi: isopentenyl pyrophosphate isomerase, IspA: farnesyl pyrophosphate synthase.

To balance the enzymatic flux and to analyze the contribution of the other five enzymes to the final yield of AD, a combinatorial approach was carried out assisted with Taguchi orthogonal array design [21], [22]. The reaction conditions were fixed at pH 7.4 and 30°C, with a constant Ads concentration of 100 mg/L (1.5 µM). The results were summarized in Table 2 and the corresponding enzyme concentrations were shown in Table S3A. Remarkably, we observed divergent AD yield with varying amounts of enzymes, where the best enzymatic ratio (run14) produced 5 fold more of AD as compared to the lowest ratio (run 5) in which the enzymes had equal activities. To further examine the influence of each enzyme on AD yield, the average effects analysis was determined. Figure 2A and 2B showed the average values of each level of the five enzymes on the AD yield. The five enzymes could be classified into two main groups: A (Erg12), B (Erg8) and D (Idi) positively enhanced AD yield when their activities were increased (Figure 2A). However, varying the activities of Erg19, and IspA did not have appreciable effect over AD yield (Figure 2B). Moreover, the half normal plot (Figure 2C) clearly indicated that Erg12, Erg8 and Idi were three main factors that had stronger influence to maximize AD yield. Therefore, among the 16 runs, higher AD yield was obtained from combinations where all the three main enzymes were at higher activities (run 14 and run 12). The model predicted the maximum AD yield would be achieved when the first four enzymes were at their highest activities (Table 2). Attempt to validate this finding showed no significant improvement over AD yield (Figure S2), suggesting that the activity ratio of Erg12:Erg8:Erg19:Idi:IspA:Ads of 100∶100∶1∶25∶5∶1 (run 14) was near the local optimum. Further improvement of AD yield may require a change in the pre-determined reaction conditions. More importantly, this combination of enzymatic levels resulted in the highest specific AD yield among all the experimental runs designed by Taguchi method. Therefore, this enzymatic ratio was chosen as the reference condition to further optimize the multienzymatic synthesis system.

**Table 2:** Taguchi L16 (4^5^) orthogonal arraray design and results.

	Levels*							
Runs	A	B		C	D	E	AD Yield	Specific AD Yield
1	2	3		4	1	2	11%	34
2	2	4		3	2	1	12%	62
3	1	2		2	2	2	9%	63
4	1	4		4	4	4	18%	16
5	1	1		1	1	1	5%	49
6	1	3		3	3	3	10%	29
7	2	1		2	3	4	8%	15
8	4	2		3	1	4	14%	24
9	3	3		1	2	4	13%	27
10	4	3		2	4	1	24%	33
11	2	2		1	4	3	16%	23
12	3	4		2	1	3	9%	39
13	3	2		4	3	1	10%	22
14	4	4		1	3	2	26%	72
15	3	1		3	4	2	13%	19
16	4	1		4	2	3	16%	32
Predicted Max AD yield 1 (P1)	4	4		4	4	2	15%	17
Predicted Max AD yield 2 (P2)	4	4		4	4	1	2%	2
Experimentally Max AD yield	4	4		1	3	2	20%	56
*Analysis of variance*								
Model p-value	0.0046							
R^2^	0.94							
Adj-R^2^	0.85							

* Refer to table S3A for the actual enzyme concentrations corresponding to the coded levels. A: mevalonate kinase (Erg12), B: phosphomevalonate kinase (Erg8), C: diphosphomevalonate decarboxylase (Erg19), D: isopentenyl pyrophosphate isomerase (Idi), E: farnesyl pyrophosphate synthase (IspA).

### Optimize IspA and Ads Levels to Enhance AD Yield

**Figure 3:**
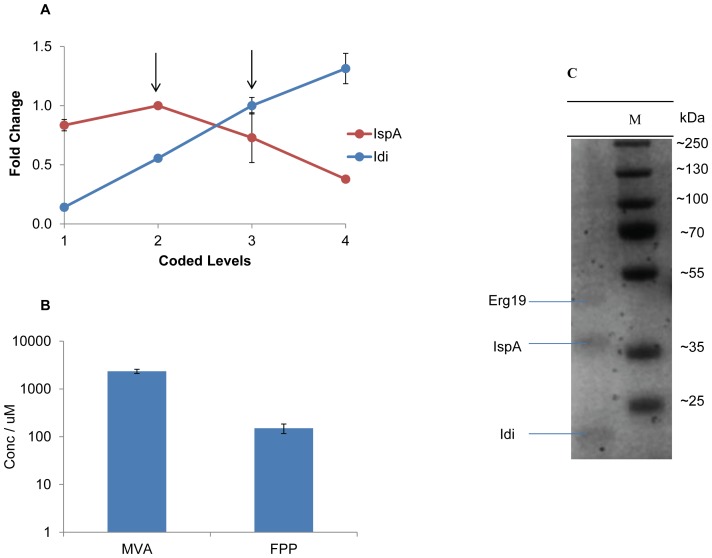
Inhibitory effect of IspA and analysis of the precipitates. A set of separate experiments was conducted to validate the inhibitory effect of IspA. This was attributed to the precipitation of FPP. A: Fold change in amorpha-4,11-diene (AD) yield when increasing IspA and Idi concentrations while keeping other enzymes at reference level. Fold change in AD yield was calculated by normalizing against AD yield obtained by reference enzyme levels, as indicated by the arrows. Presented data were average of triplicates and standard errors were drawn on the plot. B: UPLC-(TOF)MS analysis of the intermediates in the precipitates. Presented data were average of triplicates and standard errors were drawn on the plot. C: SDS-PAGE analysis of enzymes in the precipitates. The molecular weight of the each band present in the protein marker is indicated. The abbreviations are as follows. Erg19: diphosphomevalonate decarboxylase, Idi: isopentenyl pyrophosphate isomerase, IspA: farnesyl pyrophosphate synthase, MVA: mevalonic acid, FPP: farnesyl pyrophosphate.

One notable conclusion drawn from the Taguchi model prediction was that, to maximize AD yield, the activities of the first four enzymes were required to be maximized while retaining the activity of the fifth enzyme IspA, at moderate levels (Table 2). This alluded to the possibility of an inhibitory effect of IspA enzyme, since intuitively the yield should increase with increasing enzyme concentration. This led us to conduct a set of separate experiments where IspA concentrations were optimized, while retaining the other four enzymes at their reference levels. Figure 3A showed the fold change in AD yield, with respect to that obtained by the reference enzymatic levels, when either Idi or IspA concentrations were varied. Idi concentrations were optimized as a control as it displayed a positive correlation with AD yield (Figure 3A). In contrast, a remarkable inhibitory effect was found when IspA activity was increased above its reference level (Figure 3A). A critical lead at this point of the study was that precipitates were formed in the reaction when IspA activity was increased. This interesting observation led us to hypothesize that the inhibitory effect of IspA was correlated with the precipitation. Interestingly, LCMS analysis revealed that the precipitates contained FPP and MVA which are the product of IspA, and the raw material respectively (Figure 3B). SDS-PAGE indicated that enzymes were co-precipitated, and therefore, exacerbating the overall productivity of the multienzyme reaction (Figure 3C). To further identify which factor was the main reason that induced precipitation, the multienzyme reaction was analyzed stepwise. Precipitates were only visible when FPP was produced (Table S4). Therefore, it was hypothesized that the negative relationship of the increased activity of IspA and the overall productivity may be due to the accumulation of FPP in the context of the system examined. In order to test this hypothesis and further improve the AD yield of the system, response surface methodology was carried out to optimize the activities of IspA and Ads, so as to minimize the accumulation of FPP. The concentration ranges of IspA and Ads were chosen to be above and inclusive of the reference levels (Table 3 and Table S3B). The experimental data obtained based on the design was fitted to a linear mathematical model (Equation 3). The R^2^ and adjusted R^2^ values were 0.93 and 0.91 respectively (Table 3), which indicated that the model was suitable to represent adequately the real relationships between the factors used. Interpretations from the model coefficients suggested a marked agreement with previous observation that IspA level was negatively correlated with AD yield. Moreover, the model implied a positive correlation between Ads level and AD yield, and Ads had a more pronounced effect on AD production. Thus, to validate the model, Ads activity was increased twice when compared to the reference enzymatic ratio. Unexpectedly, the conversion from MVA to AD was ∼100%, which was ∼5 fold improvement of AD yield as compared to the reference condition (Figure S3). This further confirmed that Ads was the rate limiting step in the multienzyme synthesis reaction for AD production.
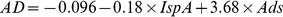
(3)


**Table 3:** Coded level combinations for a five-level, two factor response surface methodology with central composite design.

	Levels*		
Run	A	B	AD (mg/L)
1	1	5	15.9
2	3	3	8.1
3	3	3	10.4
4	5	5	15.6
5	1	1	3.6
6	0.17	3	11.9
7	5	1	3.8
8	3	0.17	0.3
9	5.8	3	9.9
10	3	3	10.7
11	3	5.8	24.9
12	3	3	9.5
*Analysis of variance*			
Model p-value	<0.0001		
R^2^	0.93		
Adj-R^2^	0.91		

* Refer to table S3B for the actual enzyme concentrations corresponding to the coded levels. A: farnesyl pyrophosphate synthase (IspA), B: amorpha-4,11-diene synthase (Ads).

### Enhancement Ads Specific Activity by Buffer Optimization

**Figure 4:**
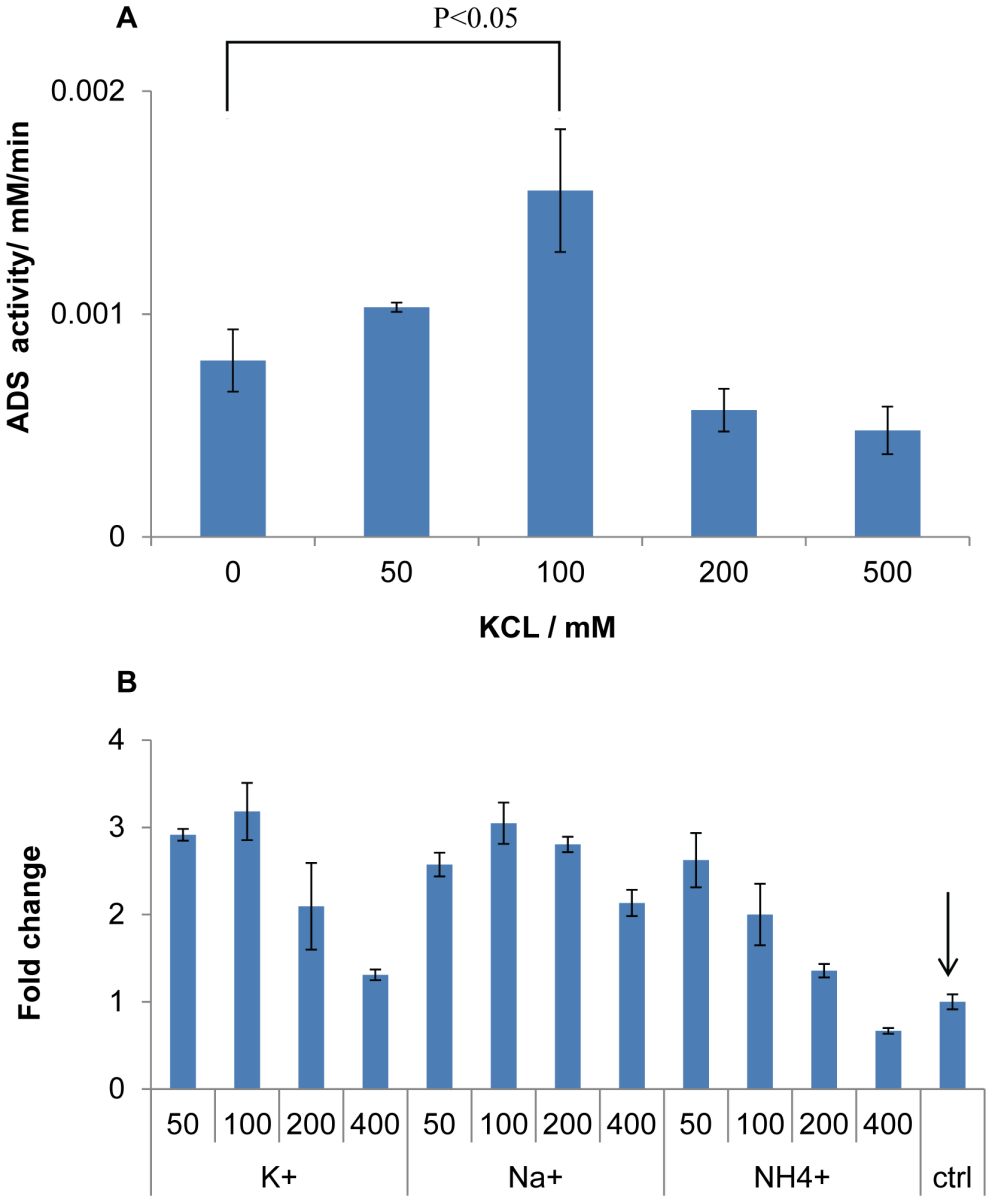
Effects of monovalent ions. Monovalent ions were used to increase the specific activity of amorpha-4,11-diene synthase (Ads) and hence the specific amorpha-4,11-diene (AD) yield of the multienzyme synthesis reaction. A: Titration of potassium chloride concentrations, and their effects on Ads specific activity. Presented data were average of triplicates and standard errors were drawn on the plots. Student’s t-Test with paired two samples for means was used to calculate the p-value in the statistical analysis. B: Titration of different monovalent ions concentrations and their effects on AD yield by reference enzymatic levels. Fold change in AD yield was calculated by normalizing against AD yield obtained by reaction without addition of monovalent ions, as indicated by the arrow. Presented data were average of triplicates and standard errors were drawn on the plots.

Next, an attempt was made to enhance AD specific yield by examining the contribution of ions in the buffer. Potassium ion has recently been shown to significantly improve the activity of a terpene synthase by interacting with the H1-α loop of the enzyme [20]. Interestingly, a similar structure was found in Ads (Figure S4). Thus, to test the effectiveness of monovalent ion, we supplemented the Ads reaction buffer with potassium ions. Figure 4A and 4B showed the change in Ads specific activity and the fold change in AD yield with respect to the reference condition respectively. As predicted, Ads specific activity was found to be enhanced approximately twice with 100 mM potassium ion (Figure 4A). More interestingly, the overall AD yield by the reference enzymatic ratio was significantly improved about three times in the presence of 100 mM potassium (Figure 4B). Other monovalent ions, sodium chloride and ammonium chloride, were also titrated into the multienzyme reaction. A similar trend was observed that, with either 100 mM sodium ion or 50 mM ammonium ion, there was a marked three-fold improvement of AD yield (Figure 4B).

**Figure 5:**
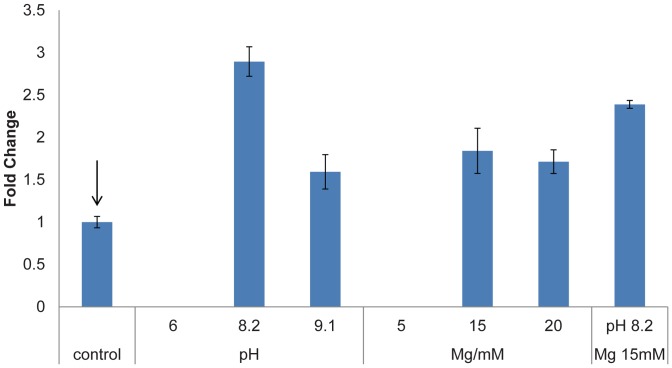
Optimization of buffer pH and magnesium concentration. Varying buffer pH and magnesium concentrations was found to be helpful to enhance the specific amorpha-4,11-diene (AD) yield. Fold change in AD yield was calculated by normalizing against AD yield obtained with buffer pH 7.4 and 10 mM Mg^2+^, as indicated by the arrow. Presented data were average of triplicates and standard errors were drawn on the plots.

To further explore the effect of the buffer used, we varied pH from 6 to 9.1 and magnesium concentrations from 5 mM to 20 mM for AD production with reference enzymatic levels. Figure 5 showed the fold change in AD yield, with respect to the reference condition, when either pH or Mg^2+^ concentrations was varied. Remarkably, AD yield increased 3 times when the pH increased from 7.4 to 8.2, and there was no amorpha-4,11-diene detected when the pH was reduced to 6. By keeping pH at 7.4, the optimum Mg^2+^ concentration was found to be 15 mM, which resulted in a moderate 1.8 fold improvement of AD yield. However, no synergistic effect was observed at pH 8.2 and 15 mM Mg^2+^. The optimum condition found was at pH 8.2 with 10 mM Mg^2+^, which significantly enhanced specific AD yield three times (Figure 5).

## Discussion

In metabolic engineering and synthetic biology, an essential process is the *in vivo* balancing of metabolic pathway flux to achieve optimal productivity. Early successes of controlling pathway flux *in vivo* have been mainly achieved by tuning the promoter strength, ribosomal binding site sequences and plasmid copy numbers [23]. Controlling enzymatic concentration and activities *in vivo* is a significant challenge where limitations in global cellular and biochemical mechanisms including the solubility of overexpressed enzymes are not easily predictable [13]. Hence, cell-free enzymatic reaction would be an enabling technology that offers an alternative to these challenges. Recently, multienzyme synthesis for therapeutic products has been successfully demonstrated [24]–[26] In this study, the mevalonate pathway together with the downstream plant enzyme amorpha-4,11-diene synthase was individually purified and reconstituted in a single vessel and the overall biochemical reaction achieved an almost complete theoretical yield (340 mg/L) of amorpha-4,11-diene production.

One prerequisite for the system is the availability of functional and purified enzymes. Most of the recombinant enzymes produced in this study were insoluble and thus pose a challenge to obtaining sufficient amounts for *in vitro* reconstitution. When Ads was overexpressed in *E. coli,* most of the enzyme was found in inclusion bodies [27]. This is possibly due to the nature of the enzyme which more than 30% of the amino acids of Ads containing hydrophobic structures, thus rendering the enzyme less soluble as compared to other pathway enzymes. Purification of terpene synthases is challenging, that some active enzymes were only recovered from inclusion bodies by *in vitro* refolding [28]–[30]. For Ads, using commercially available detergent to lyse the cells, we were unable to recover any functional enzymes. Repeated freeze-thaw method was found to be an effective method to improve the recovery of functional and soluble Ads for *in vitro* reaction.

Although the mevalonate pathway has been extensively optimized in engineered microbes, the *in vivo* mechanistic interactions of the combinations of pathway enzymes affecting amorpha-4,11-diene production are currently unknown. One advantage of the *in vitro* multienzyme system is the ability to precisely control and modulate the enzymatic activities in the same reaction condition. This allows the identification of interacting components or factors which can then guide further optimization. The Taguchi orthogonal array design methodology was used in this study to rapidly and efficiently identify the local optimal ratio of enzymatic activities and attempts to further manipulate the ratio did not result in significant improvement in the yield of AD. This approach was rather helpful in identifying the negative effect of IspA which was due mainly to the accumulation and precipitation of FPP as well as the enzymes. Attempts were made to understand the involvement of MVA in the formation of precipitates. There was no obvious precipitates formation when MVA but not FPP was present in the reaction, suggesting a stochastic process that possibly resulted from the charge interaction between high concentration of MVA and Mg^2+^. Similarly, no precipitation was found when enzymes were present without FPP. Hence, it was likely that FPP accumulated to sufficiently high levels may then precipitate, an observation consistent with a previous report [31]. FPP contains a hydrophobic 15-carbon moiety and is involved in post-translational lipid modification by protein farnesyltransferases [32], [33]. It is not unexpected that Mg^2+^ counterions may shield the negative charges of the pyrophosphate moiety resulting in the precipitation of FPP along with the enzymes. By increasing Ads activity, the flux of FPP towards AD production would have increased, and hence minimizing the accumulation of FPP. A benefit from such an increase in metabolite flux is the remarkable increase in the conversion of mevalonic acid (∼100%). It will be interesting to examine the effect of co-immobilizing IspA and Ads to improve the channeling of substrate by proximity effect in the future.

By optimizing the buffer conditions, the specific AD yield was further enhanced, demonstrating the flexibility of the *in vitro* system conditions often intolerable to cells. Monovalent ions were found to be effective in improving both the specific activity of Ads and the specific AD yield by 2 and 3 fold, respectively. Monovalent ions are well known to be required for many enzymatic activities [34]. Whether the monovalent ions may act as an allosteric activator to Ads by binding to specific secondary structures, e.g. H1-α loop of the enzyme [20], remains to be verified.

pH and magnesium ions play a critical role in moderating the enzymatic activities. Individual enzyme displayed vast different kinetic property under different pH and magnesium concentrations. Literature suggested that purified Ads displayed a higher catalytic efficiency when pH was increased in the presence of Mg^2+^ [18]. Therefore, the increased productivity of the multienzymatic pathway with increased buffer pH would likely be due to the enhanced specific activity of Ads in a more alkaline medium and the hypothesis remained to be verified.

### Conclusion

In summary, amorpha-4,11-diene was successfully produced and achieved quantitative conversion by a multienzyme, biphasic system. We have demonstrated the utility of Taguchi method to efficiently identify the local optimum enzymatic ratio and identified the inhibitory effect of IspA, resulting in the accumulation of FPP. Further optimization of IspA and Ads activities by response surface methodology identified that Ads was a critical factor. By increasing the Ads activity, almost 100% conversion from raw materials to AD has been achieved. Further buffer optimization of monovalent ions, pH and magnesium ions, was able to enhance the specific AD yield 2–3 fold significantly. The work-flow demonstrated here will be valuable to produce other isoprenoids in an efficient manner.

## Supporting Information

Figure S1 **Solubility of the pathway enzymes.**
(DOC)

Figure S2 **Validation of the Taguchi orthogonal array design.**
(DOC)

Figure S3 **Summary of optimization of amorpha-4,11-diene production.**
(DOC)

Figure S4 **Amino acid sequence alignment of H-α1 loop and Ads.**
(DOC)

Table S1 **Bacterial strains and plasmids used in this study.**
(DOC)

Table S2 **List of primers used for this study.**
(DOC)

Table S3 **Actual enzyme concentrations corresponding to the coded levels in A: Taguchi orthogonal array design, B: Response surface methodology.**
(DOC)

Table S4 **Stepwise reaction to identify the cause of precipitation.**
(DOC)
