# Supplementary material for: Statistical Experimental Design Guided Optimization of a One-Pot Biphasic Multienzyme Total Synthesis of Amorpha-4,11-diene
Source: PLoS One. 2013 Nov 20;8(11):e79650. doi: 10.1371/journal.pone.0079650 (PMC3835790; doi:10.1371/journal.pone.0079650)

**Supplementary figure S1. Solubility of the pathway enzymes.** A: solubility of the pathway enzymes measured based on the method described previously [13]. B: western blot of the supernatant and pellet of the bacterial strains overexpressing Ads, by anti-his_6_ antibody. The abbreviations are as follows. Erg12: mevalonate kinase, Erg8: phosphomevalonate kinase, Erg19: diphosphomevalonate decarboxylase, Idi: isopentenyl pyrophosphate isomerase, IspA: farnesyl pyrophosphate synthase. Ads: amorpha-4,11-diene synthase. IS: insoluble fraction. S: Soluble fraction.


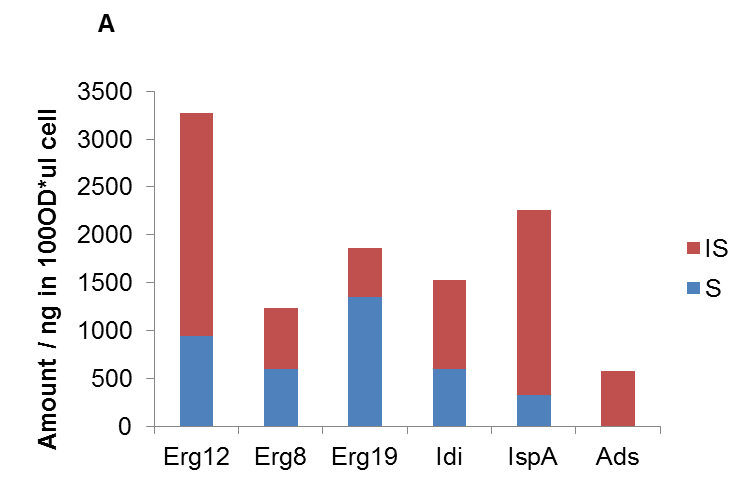


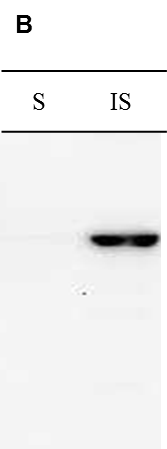

Supplement: Figure S1 — Solubility of the pathway enzymes. (DOC) [file pone.0079650.s001.doc]
