# Supplementary material for: Statistical Experimental Design Guided Optimization of a One-Pot Biphasic Multienzyme Total Synthesis of Amorpha-4,11-diene
Source: PLoS One. 2013 Nov 20;8(11):e79650. doi: 10.1371/journal.pone.0079650 (PMC3835790; doi:10.1371/journal.pone.0079650)

**Supplementary figure S2. Validation of the Taguchi orthogonal array design.** P1 and P2 represent the two predicted best enzymatic activity ratio by Taguchi method. For detailed enzymatic ratio, please refer to table 2 and supplementary table 3. Baseline represents the best enzymatic ratio obtained experimentally. One-tail student t-test for mean value was carried out and suggested that there is no statistically significant difference between the two optimal combinations (p>0.05) for amorpha-4,11-diene (AD) production. However, there is a remarkable difference between AD specific yield (p<0.0001). All the experiments were done in triplicates.


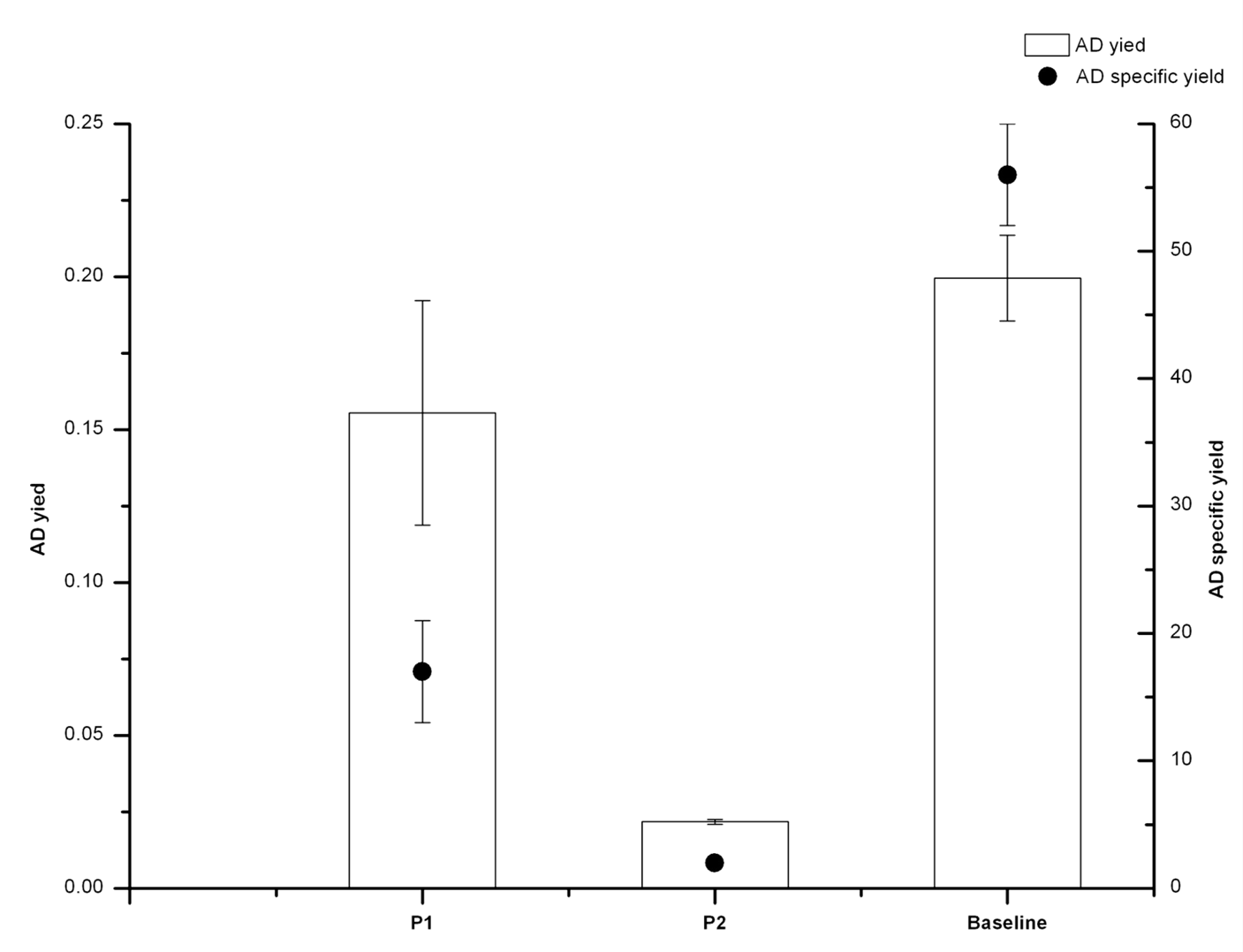

Supplement: Figure S2 — Validation of the Taguchi orthogonal array design. (DOC) [file pone.0079650.s002.doc]
