# Supplementary material for: Statistical Experimental Design Guided Optimization of a One-Pot Biphasic Multienzyme Total Synthesis of Amorpha-4,11-diene
Source: PLoS One. 2013 Nov 20;8(11):e79650. doi: 10.1371/journal.pone.0079650 (PMC3835790; doi:10.1371/journal.pone.0079650)

**Supplementary figure S3. Summary of optimization of amorpha-4,11-diene production**. EA: equal activities of the enzyme, which their concentrations in terms of Taguchi coded levels are Erg12(1), Erg8(1), Erg19(1), Idi(1), IspA(1) . TOA: optimized enzymatic activities by Taguchi orthogonal array method, which their concentrations in terms of Taguchi coded levels are Erg12(4), Erg8(4), Erg19(1), Idi(3), IspA(2). This combination of enzyme concentrations was used as the reference condition. RSM: response surface design suggested increasing Ads activity. The other five enzymes were kept at reference level.


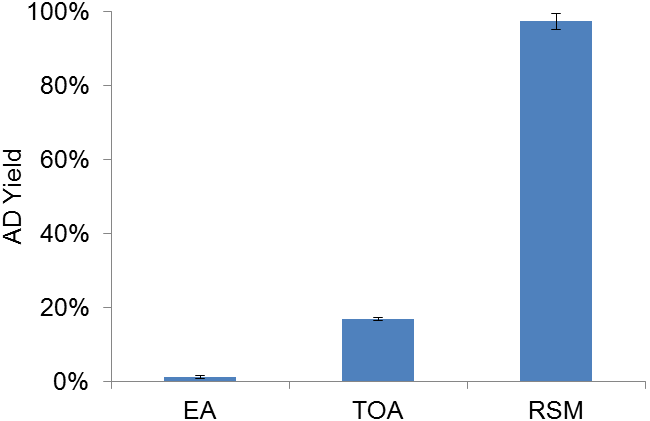

Supplement: Figure S3 — Summary of optimization of amorpha-4,11-diene production. (DOC) [file pone.0079650.s003.doc]
