# Supplementary figures and images for: Statistical Experimental Design Guided Optimization of a One-Pot Biphasic Multienzyme Total Synthesis of Amorpha-4,11-diene
Source: PLoS One. 2013 Nov 20;8(11):e79650. doi: 10.1371/journal.pone.0079650 (PMC3835790; doi:10.1371/journal.pone.0079650)

**Supplementary Figure S4. Amino acid sequence alignment of H-α1 loop [20] and Ads.**


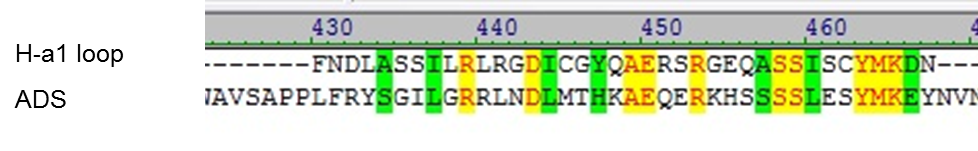

Supplement: Figure S4 — Amino acid sequence alignment of H-α1 loop and Ads. (DOC) [file pone.0079650.s004.doc]
