# Supplementary material for: Statistical Experimental Design Guided Optimization of a One-Pot Biphasic Multienzyme Total Synthesis of Amorpha-4,11-diene
Source: PLoS One. 2013 Nov 20;8(11):e79650. doi: 10.1371/journal.pone.0079650 (PMC3835790; doi:10.1371/journal.pone.0079650)
Supplement: Table S1 — Bacterial strains and plasmids used in this study. (DOC) [file pone.0079650.s005.doc]

| Name | Description | Reference |
| --- | --- | --- |
| *E. coli* BL21-Gold (DE3) | F^–^ ompT hsdS (r_B_^–^ m_B_^–^) dcm^+^ Tet^r^ gal λ(DE3) endA Hte | Stratagene |
| *E. Coli* DH10B | araD139 Δ(ara-leu)7697 fhuA lacX74 galK (Φ80 Δ(lacZ)M15) mcrA galU recA1 endA1 nupG rpsL Δ(mrr-hsdRMS-mcrBC) | NEB |
| *E. Coli* XL10-Gold | Tetr D(mcrA)183 D(mcrCB-hsdSMRmrr) 173 endA1 supE44 thi-1 recA1 gyrA96 relA1 lac Hte [F9 proAB lacIqZDM15 Tn10 (Tetr) Tn5 (Kanr) Amy] | Stratagene |
| pTrc-His_6_-Erg12 | Plasmid for overexpression of Erg12 in *E. Coli* DH10B | This study |
| pTrc-His_6_-Erg8 | Plasmid for overexpression of Erg8 in *E. Coli* DH10B | This study |
| pTrc-His_6_-Erg19 | Plasmid for overexpression of Erg19 in *E. Coli* DH10B | This study |
| pET-His_6_-Idi | Plasmid for overexpression of Idi in *E. Coli* BL21-Gold (DE3) | [13] |
| pET-His_6_-IspA | Plasmid for overexpression of IspA in *E. Coli* BL21-Gold (DE3) | [13] |
| pBAD-Ads-His_6_ | Plasmid for overexpression of Ads in E. Coli DH10B | This study |

Supplementary table S1. Bacterial strains and plasmids used in this study
