# Supplementary material for: Statistical Experimental Design Guided Optimization of a One-Pot Biphasic Multienzyme Total Synthesis of Amorpha-4,11-diene
Source: PLoS One. 2013 Nov 20;8(11):e79650. doi: 10.1371/journal.pone.0079650 (PMC3835790; doi:10.1371/journal.pone.0079650)
Supplement: Table S2 — List of primers used for this study. (DOC) [file pone.0079650.s006.doc]

| Primer Name | Sequnce |
| --- | --- |
| SacI-Sc.ERG12 Forward  Sc.ERG12-XhoI Reverse | GCGAGCTCTCATTACCGTTCTTAACTTCTGC  GCCTCGAGTTATGAAGTCCATGGTAAATTCG |
| SacI-Sc.ERG8 Forward  Sc.ERG8-XhoI Reverse | GCGAGCTCTCAGAGTTGAGAGCCTTCAGT  GCCTCGAGTTATTTATCAAGATAAGTTTCCGGA |
| SacI-Sc.Erg19 Forward  Sc.Erg19-XhoI Reverse | GCGAGCTCACCGTTTACACAGCATCCG  GCCTCGAGTTATTCCTTTGGTAGACCAGTCT |
| SacI-Ec_idi Forward  Ec_idi-XhoI Reverse | GCTTAGAGCTCCAAACGGAACACGTCA  GTAACCTCGAGTTATTTAAGCTGGGTAAATGC |
| SacI-Ec_ispA Forward  Ec_ispA-XhoI Reverse | GCTTAGAGCTCGACTTTCCGCAGCAACT  GTAACCTCGAGTTATTTATTACGCTGGATGA |
| SacI-Ads Forward  Ads-His6-XhoI Reverse | GCGGAGCTCTCTCTGACTGAGGAAAAACCA  CGCCTCGAGTCAGTGATGGTGATGATGATG |

Supplementary Table S2. List of primers used for this study.

Underlined text: restriction enzyme site
