# Supplementary material for: Statistical Experimental Design Guided Optimization of a One-Pot Biphasic Multienzyme Total Synthesis of Amorpha-4,11-diene
Source: PLoS One. 2013 Nov 20;8(11):e79650. doi: 10.1371/journal.pone.0079650 (PMC3835790; doi:10.1371/journal.pone.0079650)
Supplement: Table S3 — Actual enzyme concentrations corresponding to the coded levels in A: Taguchi orthogonal array design, B: Response surface methodology. (DOC) [file pone.0079650.s007.doc]

Supplementary table S3: Actual enzyme concentrations corresponding to the coded levels in A: Taguchi orthogonal array design, B: Response surface methodology.

A

|  | Coded levels | | | |
| --- | --- | --- | --- | --- |
| Variables (mg/L) | 1 | 2 | 3 | 4 |
| A: Erg12 | 0.8 | 4 | 20 | 80 |
| B: Erg8 | 0.2 | 1 | 5 | 20 |
| C: Erg19 | 1.5 | 7.5 | 37.5 | 150 |
| D: Idi | 1.8 | 9 | 45 | 180 |
| E: IspA | 1.8 | 9 | 45 | 180 |

B

|  | Coded levels | |
| --- | --- | --- |
| Variables (mg/L) | 1 | 5 |
| A: IspA | 36 | 180 |
| B: Ads | 36 | 180 |
| Alpha (Rotatable) | 1.41 |  |
