# Supplementary material for: Statistical Experimental Design Guided Optimization of a One-Pot Biphasic Multienzyme Total Synthesis of Amorpha-4,11-diene
Source: PLoS One. 2013 Nov 20;8(11):e79650. doi: 10.1371/journal.pone.0079650 (PMC3835790; doi:10.1371/journal.pone.0079650)
Supplement: Table S4 — Stepwise reaction to identify the cause of precipitation. (DOC) [file pone.0079650.s008.doc]

Supplementary table S4: Stepwise reaction to identify the cause of precipitation

| Reaction | Precipitation | Estimated concentration of FPP |
| --- | --- | --- |
| Erg12-Erg8-Erg19 | No | - |
| Erg12-Erg8-Erg19-Idi-IspA | Yes | 0.4mM |
| Erg12-Erg8-Erg19-Idi-IspA-Ads | Yes | 0.4mM |
